# Progressive Ankylosis Protein (ANK) in Osteoblasts and Osteoclasts Controls Bone Formation and Bone Remodeling

**DOI:** 10.1002/jbmr.60

**Published:** 2010-02-08

**Authors:** Hyon Jong Kim, Takeshi Minashima, Edward F McCarthy, Jeffrey A Winkles, Thorsten Kirsch

**Affiliations:** 1 Musculoskeletal Research Center, Department of Orthopaedic Surgery, New York University Hospital for Joint DiseasesNew York, NY, USA; 2 Department of Surgery, Center for Vascular and Inflammatory Diseases, University of Maryland School of MedicineBaltimore, MD, USA; 3 Department of Pathology, The Johns Hopkins HospitalBaltimore, MD, USA

**Keywords:** Ank, osteoblast differentiation, osteoclast differentiation, skeletal phenotype, transcription factors

## Abstract

The progressive ankylosis gene (*ank*) encodes a transmembrane protein that transports intracellular inorganic pyrophosphate (PP_*i*_) to the extracellular milieu. *ank*/*ank* mice, which express a truncated nonfunctional ANK, showed a markedly reduced bone mass, bone-formation rate, and number of tartrate-resistant acid phosphatase–positive (TRAP^+^) multinucleated osteoclasts. ANK function deficiency suppressed osteoblastic differentiation of *ank/ank* bone marrow stromal cells, as indicated by the decrease in the expression of bone marker genes, including *osterix*, reduced alkaline phosphatase activity, and mineralization. *Runx2* gene expression levels were not altered. Conversely, overexpression of ANK in the preosteoblastic cell line MC3T3-E1 resulted in increased expression of bone marker genes, including *osterix*. Whereas *runx2* expression was not altered in ANK-overexpressing MC3T3-E1 cells, *runx2* transcriptional activity was increased. Extracellular PP_*i*_ or P_*i*_ stimulated osteoblastogenic differentiation of MC3T3-E1 cells or partially rescued delayed osteoblastogenic differentiation of *ank/ank* bone marrow stromal cells. A loss of PP_*i*_ transport function ANK mutation also stimulated osteoblastogenic differentiation of MC3T3-E1 cells. Furthermore, ANK function deficiency suppressed the formation of multinucleated osteoclasts from *ank/ank* bone marrow cells cultured in the presence of macrophage colony-stimulating factor and receptor activator of nuclear factor-κB ligand. In conclusion, ANK is a positive regulator of osteoblastic and osteoclastic differentiation events toward a mature osteoblastic and osteoclastic phenotype. © 2010 American Society for Bone and Mineral Research.

## Introduction

Bone is a dynamic organ that undergoes continuous remodeling, in which bone resorption by osteoclasts precedes bone formation by osteoblasts. A proper balance between bone resorption and bone formation is required to maintain bone mass and integrity. Impaired bone formation, resorption, or both results in either decreased bone mass, as observed in osteoporosis, or increased bone mass, as observed in osteopetrosis and osteosclerosis.(1,2) Osteoblasts arise from mesenchymal stem cells that can differentiate into adipocytes, chondrocytes, myoblasts, and osteoblasts. The differentiation of multipotential mesenchymal stem cells into osteoblasts is controlled by two transcription factors, runx2 and osterix. Both *runx2-* and *osterix*-null mice show a complete absence of endochondral and intramembranous bone formation owing to a lack of osteoblast differentiation.(3–6) *Osterix* has been shown to act downstream of *runx2*, and it has been suggested that *runx2* regulates early differentiation of mesenchymal cells into preosteoblastic cells, whereas *osterix* controls the differentiation of preosteoblastic cells into immature osteoblasts.(6,7) In addition, it has been suggested that *runx2* regulates *osterix* expression.(8)

Bone resorption involves both dissolution of bone mineral and degradation of the organic bone matrix. Both functions are performed by osteoclasts. Osteoclasts are members of the monocyte/macrophage lineage and are formed by multiple instances of cellular fusion of their mononuclear precursors. Monocytes can be induced to differentiate into osteoclasts in the presence of receptor activator of nuclear factor-κB (RANK) ligand (RANKL) and macrophage colony-stimulating factor (M-CSF). RANKL and M-CSF are expressed by osteoblasts. Osteoclast precursor cells express RANK, which is the receptor for RANKL.(9–11) In addition, osteoblasts express a decoy receptor for RANK, osteoprotegrin (OPG), that binds to RANKL and inhibits its binding to RANK. RANKL/RANK signaling activates four pathways that mediate osteoclast formation [nuclear factor-κB (NF-κB), c-fos, and calcineurin/NFATc1] and three pathways that mediate osteoclast activation (Src and MKK6/p38/MITF) and survival (Src and extracellular signal–regulated kinase).(9)

The progressive ankylosis gene (*ank*) encodes a transmembrane protein that transports intracellular inorganic pyrophosphate (PP_*i*_) to the extracellular milieu.(12) Recently, human mutations in *ank* have been discovered that lead to craniometaphyseal dysplasia (CMD).(13,14) CMD mutations have been identified in the human *ank* gene in the form of point mutations and one-amino-acid insertions or deletions that cluster mostly in cytoplasmic domains close to the C-terminus. The main phenotypes of the disease are progressive thickening of craniofacial bones and flaring metaphyses with increased radiolucency of long bones.(15) In addition, histopathologic studies of CMD patients showed either increased osteoblast numbers, no osteoclasts in periosteal or endosteal layers, or increased osteoblast and osteoclast numbers.(16–19) Furthermore, a knock-in mouse model for CMD expressing a human mutation (Phe377 deletion) in ANK shows defects in bone formation and bone remodeling.(20) Altogether these findings suggest that ANK plays a regulatory role in bone formation and resorption and may directly affect osteoblast and osteoclast differentiation and/or function. Nevertheless, the exact role of ANK in these processes is not understood.

A spontaneous mutation in the murine *ank* gene resulted in a premature stop codon and the expression of a nonfunctional protein. The lack of ANK function in these *ank/ank* mice caused generalized arthritis associated with extensive hydroxyapatite deposition in articular cartilage and synovial fluid. The presence of ectopic joint mineral formation in these mice eventually leads to complete fusion and immobility of almost every joint. In addition, these mice exert spinal, peripheral joint, and ligament bony ankylosis and calcification of arteries.(12) A homozygous *ank*-null mouse model shows the same phenotype.(21) The ANK function–deficient *ank/ank* mice were used in this study to gain insights into the role of ANK in osteoblast and osteoclast differentiation and function. Here we report that ANK function deficiency suppressed bone formation as well as bone resorption by directly affecting osteoblast and osteoclast differentiation. Consequently, ANK function deficiency resulted in reduced numbers of mature osteoblasts and osteoclasts. These findings suggest that ANK is a novel regulator of bone remodeling.

## Materials and Methods

### Bone histomorphometry and micro–computed tomographic (µCT) analysis

The *ank*/*ank* breeding colony used was originally on a hybrid background derived from crossing a C3H and C57BL/6 hybrid male with a BALB/c female. Heterozygote breeders were used to generate and study *ank/ank* and wild-type littermates, with genotypes analyzed by polymerase chain reaction (PCR), as described previously.(12) Protocols were approved by the Institutional Animal Care and Use Committee at New York University School of Medicine in accordance with the National Institutes of Health Guide for the Care and Use of Laboratory Animals. Mice were euthanized at 4 weeks, 2 months, and 4 months of age. For bone histomorphometric analysis, hindlimbs from five male *ank/ank* mice and five male wild-type littermates were used. Dissected tibias from *ank*/*ank* mice and wild-type littermates were fixed in 4% paraformaldehyde, decalcified in 0.2M EDTA (pH 7.4) for 14 days, and embedded in paraffin. Then 8-µm sections were cut, stained with hematoxylin and eosin, and analyzed by bone histomorphometry. Quantitative histomorphometry was performed in an area 175 to 875 µm distal to the growth plate using OsteoMeasure software (Osteometrics, Inc., Decatur, GA, USA) in an epifluorescence microscopic system, as detailed elsewhere.(22) Images were acquired with a microscope (Eclipse 50i, Nikon, Melville, NY, USA) with ×4 or ×10 objectives (Nikon), and a digital camera with a ×0.7 reduction lens (Sony Color Video Camera 3CCD, New York, NY, USA) was used for photography. The following parameters of bone remodeling were estimated: trabecular bone volume as a percentage of total tissue volume, trabecular thickness (in µm), trabecular number (per µm), and trabecular separation (in µm).(23) On decalcified sections, tartrate-resistant acid phosphatase–positive (TRAP^+^) multinucleated cells were counted as osteoclasts to evaluate osteoclast number/bone surface and osteoclast surface/bone surface. For dynamic bone histomorphometry, tetracycline hydrochloride (25 mg/kg) injection was followed by the same dose of calcein 7 days later, and animals were euthanized 2 days after that. Histomorphometric analysis of undecalcified sections of the proximal tibial metaphysis was performed by transmitted and epifluorescent microscopy using a microscope (Olympus IX71, Olympus America Inc., Center Valley, PA, USA) and OsteoMeasure analyzing software. Single-labeled (sLS/BS) and double-labeled (dLS/BS) surface, mineralizing surface (MS; dLS + sLS/2)/BS), interlabel thickness (Ir.L.Th), and mineral apposition rate (Ir.L.Th/Ir.L.t = MAR) were measured and calculated. The interlabel time (Ir.L.t.) was 7 days. Bone-formation rate with bone surface as the referent (BFR/BS) was calculated as MAR × MS (µm^3^/µm^2^/day). Femurs and tibias from 4-week-old male *ank/ank* (*n* = 5) and male wild-type littermates (*n* = 5) were analyzed using µCT (Numira Biosciences, Salt Lake City, UT, USA). Trabecular measurements of femurs and tibias were taken at the distal growth plate in 80 consecutive slices of 12-mm resolution over a distance of 960 mm. The µCT bone analysis was performed by Numira Biosciences.

### Mouse bone marrow stromal cell culture

Bone marrow stromal cells (BMSCs) were isolated from femurs of 4-week-old *ank/ank* mice or wild-type littermates and cultured at 2 × 10^6^ cells per 10-cm^2^ well in α-minimal essential medium (α-MEM) supplemented with 15% fetal calf serum, as described previously.(24,25) After cells reached confluence, they were cultured in the presence of 5 mM β-glycerophosphate, 50 µg/mL of ascorbate, and 10^−7^ M dexamethasone to induce osteoblastogenic differentiation for up to 16 days, as described previously.(24,25) After 6 days of culture, cells were stained for alkaline phosphatase (APase) activity using alkaline phosphatase magenta immunohistochemical substrate solution (Sigma Chemical Co., St. Louis, MO, USA). An APase^+^ colony was defined as a colony that stained for APase activity and contained at least 20 cells. To determine mineralization in these cultures, von Kossa staining was performed after 16 days. A von Kossa^+^ colony was defined as a colony that reacted to von Kossa stain and contained at least 20 cells.

### MC3T3-E1 cell culture

The preosteoblastic cell line MC3T3-E1 was cultured at confluence in Dulbecco's modified Eagle's medium with 10% fetal calf serum and then cultured in the presence of ascorbate (50 µg/mL) and β-glycerophosphate (10 mM) to induce collagenous matrix release and mineralization (osteogenic differentiation medium).

### Bone marrow cell cultures

Total bone marrow cells were flushed out from *ank/ank* and wild-type femurs or tibias and were cultured overnight in tissue culture dishes in α-MEM containing 10% fetal calf serum. Nonadherent bone marrow cells were plated at a density of 1 × 10^5^ per 24-well plate in α-MEM containing 30 ng/mL of M-CSF. After 3 days, cultures were washed once with PBS and adherent cells were used as M-CSF-dependent macrophages (MDMs). MDMs were induced to undergo osteoclast differentiation with 30 ng/mL of M-CSF and 100 ng/mL of RANKL (R&D Systems, Minneapolis, MN, USA) for 4 to 6 days. Medium and cytokines were changed every other day. This method has been described to be very efficient in removing BMSCs.(26,27) TRAP^+^ multinucleated (>3 nuclei) cells were counted as osteoclasts.

### Transfections and luciferase reporter assays

MC3T3-E1 cells were transfected with empty pcDNA expression vector or pcDNA expression vector containing wild-type *ank* cDNA (pcDNA-*ank*) or the CMD mutant *ank* (F376del) cDNA (pcDNA-F376del) using Fugene 6 transfection reagent from Roche (Branchburg, NJ, USA) following the manufacturer's instructions. After transfection, cells were cultured for up to 8 days in differentiation medium. For luciferase assays, cells were cotransfected with empty pcDNA, pcDNA-*ank*, or pcDNA-F376del, a firefly luciferase reporter construct containing six *runx2* DNA-binding elements (pOSE2-luc), and a wild-type *Renilla* luciferase control reporter vector (Promega, Madison, WI, USA) using Fugene 6 transfection reagent according to the manufacturer's directions. After 48 hours, the cells were rinsed in PBS and lysed in 1× passive lysis buffer (Promega). Cell extracts were used to measure luciferase activity based on the Dual Luciferase Reporter Assay System (Promega), and the values were normalized against the efficiency of transfection using the same system. Both firefly and *Renilla* luciferase activity were measured by a Berthold luminometer (Tristar LB 941, Berthold Technologies, Oak Ridge, TN, USA). All experiments were performed in triplicate and repeated three to five times.

### Real-time PCR analysis

Total RNA was isolated from cell cultures using the RNeasy Minikit (Qiagen, Valencia, CA, USA). Gene expression was quantified by real-time PCR analysis, as described previously.(28) Briefly, 1 µg of total RNA was reverse-transcribed using the Omniscript RT kit (Qiagen). A 1:100 dilution of the resulting cDNA was used as a template to quantify the relative content of mRNA by real-time PCR using SYBR Green. The 18S rRNA was amplified at the same time and used as an internal control. The cycle threshold values for 18S rRNA and the samples were measured and calculated by computer software. Relative transcription levels were calculated as *x* = 2^–ΔΔCT^, in which ΔΔCT = ΔE – ΔC, ΔE – CT_exp_ – CT_18S_, and ΔC = CT_crtl_ – CT_18S_. The gene-specific primers used are as follows: *ank*-forward, 5-GCC CAT TGT CAA CCT CTT CGT-3, and *ank*-reverse, 5-GAA TGG CCA CTG CCT CTG TAG-3; *APase*-forward, 5-AAC ACC AAT GTA GCC AAG-3, and *APase*-reverse, 5-TCG GGC AGC GGT TAC TGT-3; *bone sialoprotein* (*BSP*)–forward, 5-TTG AGT TAG CTG CAC TCC AAC TG-3, and *BSP*-reverse, 5-CGT CGC TTT CCT TCA CTT TTG-3; *COL1A1*-forward, 5-CGA AGG CAA CAG TCG ATT CA-3, and *COL1A1*-reverse, 5-CCC CAA GTT CCG GTG TGA-3; *osteocalcin* (*OC*)-forward, 5-CCA GCG ACT CTG AGT CTG ACA A-3, and *OC*-reverse, 5-CCG GAG TCT ATT CAC CAC CTT ACT-3; *osterix*-forward, 5-TTC TGT CCC CTG CTC CTT CTA G-3, and *osterix*-reverse, 5-CGT CAA CGA CGT TAT GCT CTT C-3; *runx2*-forward, 5-AGT AGC CAG GTT CAA CGA TCT GA-3, and *runx2*-reverse, 5-GAC TGT TAT GGT CAA GGT GAA ACT CTT-3; *c-fos*-forward, 5-CCG TGT CAG GAG GCA GAG C-3, and *c-fos* reverse 5-GCA GCC ATC TTA TTC CGT TCC C-3; *Nfatc1*-forward, 5-AGC CCA AGT CTC ACC ACA GG-3, and *Nfatc1*-reverse, 5-CAG CCG TCC CAA TGA ACA GC-3; *RANK*–forward, 5 -TCG TCC ACA GAC AAA TGC AAA C-3, and *RANK*-reverse, 5-TGG AAG AGC TGC AGA CCA CAT-3; *TRAP*-forward, 5-CAC TCC CAC CCT GAG ATT TGT G-3, and *TRAP*-reverse, 5-ACG GTT CTG GCG ATC TCT TTG-3; *RANKL*-forward, 5-TTT GCA CAC CTC ACC ATC AAT G -3, and *RANKL*-reverse 5-TTA GAG ATC TTG GCC CAG CCT C-3; *osteoprotegrin* (*OPG*)-forward 5-AAG AGC AAA CCT TCC AGC TGC-3, and *OPG*-reverse 5-CGC TGC TTT CAC AGA GGT CAA-3.

### Intracellular PP_*i*_ measurements

Intracellular PP_*i*_ was measured with a spectrophotometric method using PP_*i*_-dependent fructose-6-phosphate kinase activity coupled with the disappearance of NADH (pyrophosphate reagent, Sigma). Cell layers were extracted with 0.5 mL of 1 M perchloric acid for 2 hours on ice and neutralized with 0.5 mL of 1 M KOH, and samples were centrifuged to remove the precipitate. Next, 0.4 mL of sample was added to 0.8 mL of reconstituted PP_*i*_ reagent (Sigma) and incubated for 10 minutes at 30°C. The absorption was measured at 340 nm. PP_*i*_ concentrations were determined according to the manufacturer's instructions.

### Statistical analysis

Numerical data are presented as mean ± SD (*n* ≥ 3). Statistical analysis was performed by Student's *t* test to evaluate differences between the two groups. Analysis of variance was performed when the examined experimental groups exceeded three. Tukey's multiple-comparison test was applied as a post hoc test. Statistical significance was defined as *p* < .05 (*p* values are reported in the figure legends).

## Results

### Low bone mass, reduced bone-formation rate, and reduced number of osteoclasts in tibias and femurs of *ank*/*ank* mice

**Fig. 1:**
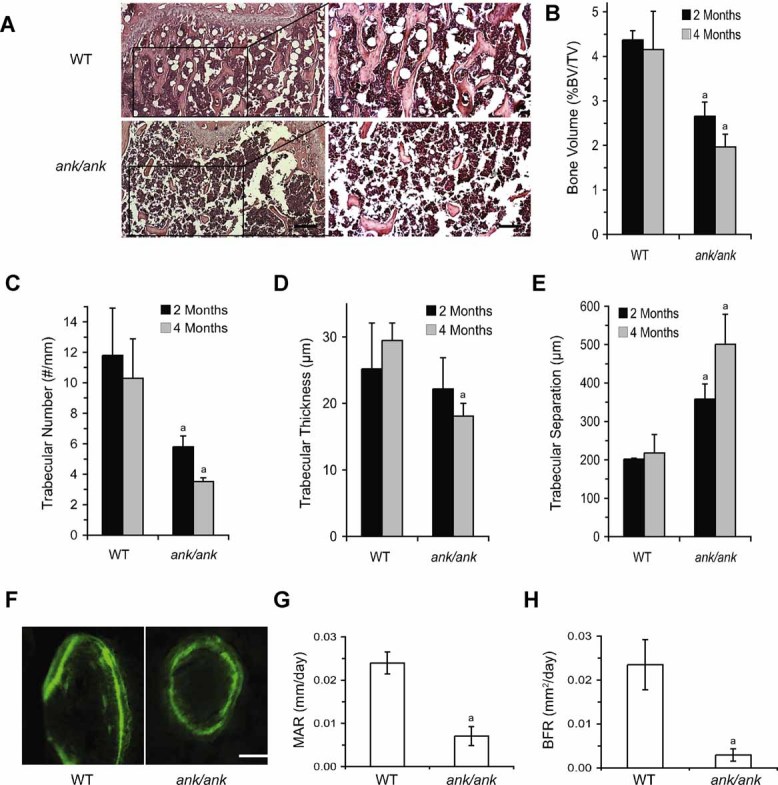
Bone mass and formation are decreased in *ank/ank* mice. (*A*) Hematoxylin and eosin staining of the proximal tibias of 4-month-old wild-type (WT) and *ank/ank* mice *(ank/ank*). Inset boxes indicate the regions of the figures to the right. Bars = 50 µm (*left*) and 200 µm (*right*). (*B–E*) Quantitative histomorphometry of proximal tibias of 2- and 4-month-old *ank/ank* mice and wild-type littermates show significantly lower bone volume/total volume and trabecular number as well as increased trabecular separation in *ank*/*ank* mice compared with wild-type littermates. Trabecular thickness was decreased significantly in 4-month-old *ank*/*ank* mice but not in 2-month-old *ank*/*ank* mice. Data are expressed as means ± SD for five male mice per genotype group (^a^*p* < .01 versus wild-type littermates). (*F*) Representative tetracycline and calcein double labelings. Bar = 200 µm. (*G, H*) Single-labeled (sLS/BS) and double-labeled (dLS/BS) surface, mineralizing surface [MS; dLS + sLS/2)/BS], interlabel thickness (Ir.L.Th), and mineral apposition rate (Ir.L.Th/Ir.L.t = MAR) were measured and calculated. The interlabel time (Ir.L.t) was 7 days. Bone-formation rate with bone surface as the referent (BFR/BS) was calculated as MAR × MS (µm^3^/µm^2^/day). Data are expressed as means ± SD for five male mice per genotype group (^a^*p* < .01 versus wild-type littermates).

Histomorphometric analysis of long bones of *ank*/*ank* mice and wild-type littermates revealed a low-bone-mass phenotype in 2- and 4-month-old *ank*/*ank* mice. Trabecular bone volume in the proximal tibial metaphysis was reduced by more than 40% in 2-month-old *ank*/*ank* mice and more than 50% in 4-month-old *ank*/*ank* mice relative to wild-type littermates (Fig. 1*A, B*). This decreased bone volume was associated with a decrease in trabecular number and thickness and a significant increase in the mean distance between individual trabeculae (Fig. 1*C–E*). Dynamic histomorphometry showed abundant double tetracycline and calcein labels on the endocortical surface of wild-type mouse tibias. In contrast, primarily single labeling and few areas of double labels with reduced distance between the tetracycline and calcein labels were observed in *ank/ank* mice. Accordingly, parameters of new bone formation (MAR and BFR) were significantly higher in wild-type than in *ank/ank* mice (Fig. 1*F–H*).

**Fig. 2:**
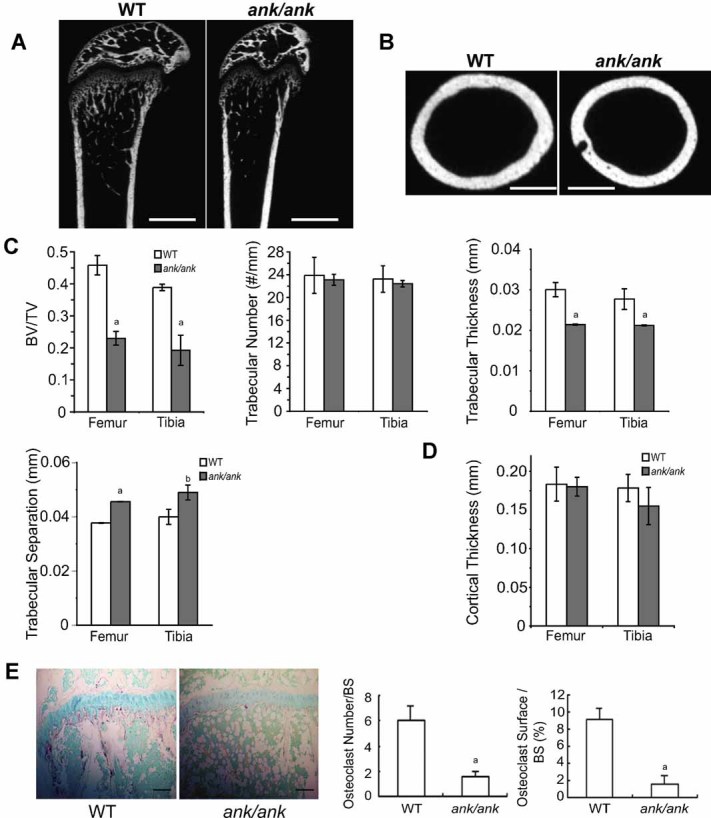
µCT analysis and TRAP staining of femurs and tibias of 4-week-old *ank/ank* mice and wild-type littermates. (*A*) 2D µCT reconstruction of the sagittal plane of the femoral metaphysis of wild-type (WT) and *ank/ank* mice. Bar = 1 mm. (*B*) 2D µCT reconstruction of the axial plane of the femoral metaphysis of wild-type and *ank/ank* mice. Bar = 1 mm. (*C*) Trabecular bone volume/tissue volume (BV/TV), trabecular number, trabecular thickness, and trabecular separation were calculated. (*D*) Cortical thickness was calculated. Cortical thickness in each sample was obtained at three points around the perimeter of each bone. Data presented in panels *C* and *D* are presented as mean ± SD for five male mice in each genotype group (^a^*p* < .01 versus wild-type littermates; ^b^*p* < .05 versus wild-type littermates). (*E*) TRAP staining of sections of tibias of 4-week-old *ank/ank* mice and wild-type littermates. Bar = 200 µm. The osteoclast number is expressed per millimeter of bone surface; osteoclast surface is expressed as percent of bone surface. Data are presented as mean ± SD. for five male mice in each genotype group (^a^*p* < .01 versus wild-type littermates).

To further characterize the bone phenotype of *ank/ank* mice, we analyzed the femurs and tibias of 4-week-old *ank/ank* and wild-type mice by µ-CT. Trabecular bone volume of the femurs and tibias was reduced in 4-week-old *ank/ank* mice compared with wild-type littermates (Figs. 2*A, C*). In addition, trabecular thickness was reduced in the femurs and tibias of 4-week-old *ank/ank* mice, whereas trabecular separation was increased in *ank/ank* femurs and tibias (Fig. 2*C*). Trabecular numbers in 4-week-old *ank/ank* femurs and tibias were similar to those in wild-type femurs and tibias (Fig. 2*C*). There was no difference between the cortical thickness of *ank/ank* and wild-type femurs and tibias (Figs. 2*B, D*). Decreased numbers of TRAP^+^ cells were detected in the metaphysis of *ank/ank* femurs and tibias compared with wild-type littermates (Fig. 2*E*). Quantitative analysis revealed a marked reduction in the number and surface area of TRAP^+^ osteoclasts in *ank/ank* femurs and tibias, suggesting that bone resorption was also reduced in *ank/ank* mice (Fig. 2*E*).

### Impaired osteoblastogenic differentiation and mineralization of bone marrow stromal cells (BMSCs) and calvarial osteoblasts isolated from *ank*/*ank* mice

**Fig. 3:**
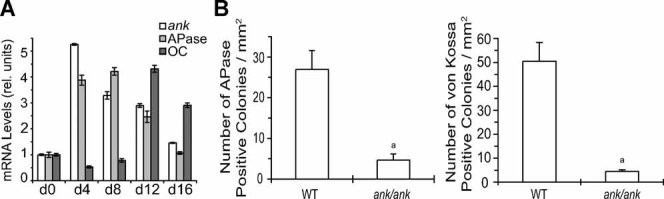
Osteoblastogenic differentiation of *ank/ank* and wild-type (WT) BMSCs. BMSCs were cultured in osteoblastogenic differentiation medium for up to 16 days. (*A*) *ank, APase*, and *osteocalcin* (*OC*) mRNA levels during osteoblastogenic differentiation of wild-type BMSCs cultured in osteoblastogenic differentiation medium were determined by real-time PCR analysis using SYBR Green and normalized to the 18S RNA levels. Data were obtained from triplicate PCRs using RNA from three different cultures, and values are presented as means ± SD. (*B*) APase activity staining was performed after 6 days in culture and von Kossa staining after 16 days in culture. An APase^+^ or von Kossa^+^ colony was defined as one that reacted to its respective stain and contained at least 20 cells. Data were obtained from four different cell cultures and are expressed as means ± SD (^a^*p* < .01 versus wild type).

**Fig. 4:**
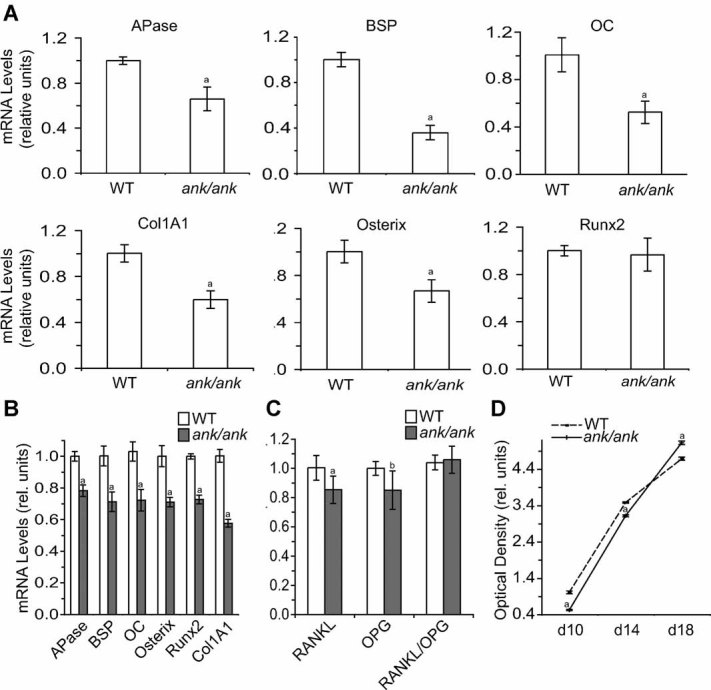
Bone marker and transcription factor gene expression levels and mineralization in *ank/ank* and wild-type (WT) BMSCs and calvarial osteoblasts. (*A, B*) The levels of bone marker mRNAs, including *APase, BSP, osteocalcin* (*OC*), *osterix, runx2*, and *type I collagen* (*Col1A1*), of wild-type and *ank/ank* BMSCs cultured for 10 days in osteoblastogenic differentiation medium (*A*) and wild-type and *ank/ank* calvarial osteoblasts (*B*) and the mRNA levels of *RANKL* and *OPG* of wild-type and *ank/ank* calvarial osteoblasts (*C*) were determined by real-time PCR and SYBR Green and normalized to 18S RNA levels. Data were obtained from triplicate PCRs using RNA from three different cultures, and values are presented as means ± SD (^a^*p* < .01 versus WT; ^b^*p* < .05 versus WT). (*D*) The degree of mineralization in wild-type and *ank/ank* calvarial osteoblasts was determined using alizarin red S staining. To quantitate the alizarin red S stain, each dish was incubated with cetylpyridinium chloride for 1 hour. The optical density of alizarin red S stain released into solution was measured at 570 nm, normalized to total amount of protein, and expressed as relative units compared with the optical density per milligram of protein of WT cells after a 10-day culture period, which was set as 1. Data were obtained from four different experiments, and values are presented as means ± SD (^a^*p* < .01 versus WT).

We isolated BMSCs from *ank*/*ank* mice and wild-type littermates and compared their ability to differentiate into mature mineralizing osteoblasts. Freshly isolated BMSCs were cultured in the presence of β-glycerophosphate, ascorbate, and dexamethasone to induce osteoblastogenic differentiation in these cells, as described previously.(24) *ank* mRNA levels were the lowest on day 0 of wild-type BMSC cultures in osteoblastogenic differentiation medium. *ank* mRNA levels increased in wild-type BMSCs when cultured in osteoblastogenic differentiation medium and reached their highest levels on day 4 (Fig. 3*A*). *APase* mRNA levels, an early marker of osteoblastic differentiation, increased on day 4 and reached the highest levels between days 4 and 8. *APase* mRNA levels declined with the onset and progression of mineralization (Fig. 3*B*). mRNA levels of *osteocalcin*, which is a late marker of osteoblast differentiation and increases during the onset of mineralization, were low up to day 8 in wild-type BMSC cultures and increased thereafter, reaching the highest level on day 12 (Fig. 3*A*). After 6 days, cultures were stained for APase activity. The number of APase-positive colonies in *ank/ank* BMSCs was markedly reduced compared with those of APase-positive colonies in wild-type BMSCs (Fig. 3*B*). Von Kossa staining after 16 days of culture was used to determine mineralized colonies in these cultures. The number of von Kossa^+^ colonies was markedly reduced in cultures of BMSCs isolated from *ank*/*ank* mice compared with those from wild-type mice (Fig. 3*B*). Real-time PCR analysis revealed that the mRNA levels of bone marker genes, including *APase, bone sialo protein* (*BSP*), *osteocalcin*, and *type I collagen*, were reduced in *ank/ank* BMSCs that had been cultured for 10 days in osteoblastogenic differentiation medium compared with the mRNA levels of these genes in wild-type BMSCs (Fig. 4*A*). The mRNA level of *osterix* also was markedly downregulated in *ank/ank* BMSCs, whereas the mRNA level of *runx2* in *ank/ank* BMSCs was similar to that in wild-type BMSCs (Fig. 4*A*). Furthermore, mRNA levels for bone marker genes, including *APase, BSP, osteocalcin, osterix, runx2*, and *type I collagen*, were decreased in *ank/ank* calvarial osteoblasts compared with the levels in wild-type calvarial osteoblasts (Fig. 4*B*). mRNA levels of *RANKL* and *OPG* also were decreased in *ank/ank* calvarial osteoblasts compared with the levels in wild-type cells; however, the *RANKL*/*OPG* ratio was similar in *ank/ank* and wild-type calvarial osteoblasts (Fig. 4*C*). Primary *ank/ank* calvarial osteoblasts demonstrated decreased matrix mineralization during the onset and early propagation phase of mineralization compared with the mineralization levels of wild-type cells, whereas at later stages of mineralization *ank/ank* calvarial osteoblasts showed increased mineralization (Fig. 4*D*). These findings suggest that osteoblastogenic differentiation is delayed in *ank/ank* BMSCs and calvarial osteoblasts. However, once mineralization is initiated, *ank/ank* calvarial osteoblast matrix mineralization is increased because of the lack of extracellular PP_*i*_ to inhibit extracellular matrix mineralization.

### Overexpression of ANK or CMD mutant ANK (F376del) enhances osteoblastogenic differentiation and *runx2* transcriptional activity

**Fig. 5:**
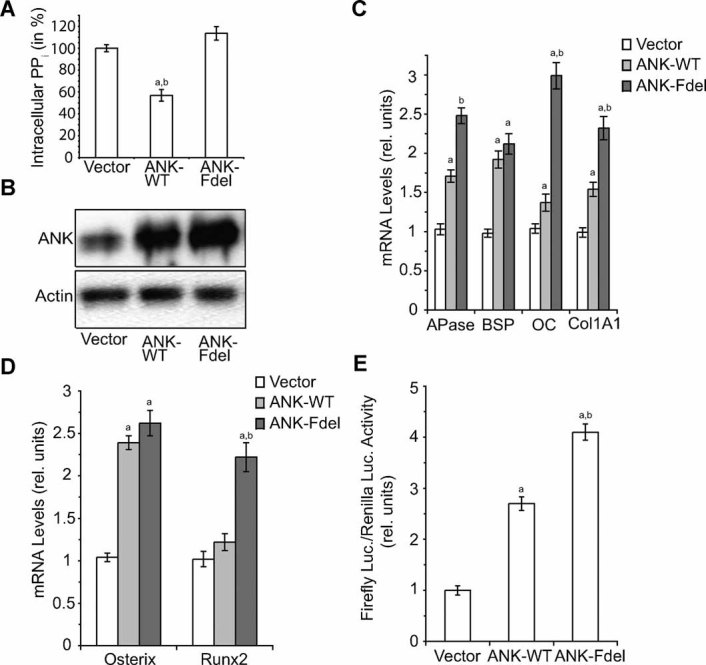
Enhanced osteoblastogenic differentiation and *runx2* transcriptional activity of MC3T3-E1 cells overexpressing wild-type ANK or the F376del mutant form of ANK. (*A*) Intracellular PP_*i*_ in COS cells transfected with empty expression vector (Vector) or vector containing wild-type *ank* (ANK-WT) or F376del *ank* (ANK-Fdel) cDNA. Intracellular PP_*i*_ concentration was assayed after 3 days of transfection. The intracellular PP_*i*_ concentration of COS cells transfected with empty vector was set as 100%. Data were obtained from three different experiments and are expressed as means ± SD (^a^*p* < .01 versus empty vector–transfected COS cells; ^b^*p* < .01 versus F376del-transfected COS cells). (*B*) Expression of ANK protein in MC3T3-E1 cells transfected with empty expression vector (Vector) or expression vector containing wild-type *ank* cDNA (ANK-WT) or F376del *ank* cDNA (ANK-Fdel) was analyzed by immunoblotting with an antibody specific for ANK. Immunoblotting with an antibody specific for actin was performed to demonstrate equal loading of the gel and to show that transfection of MC3T3-E1 cells affects only ANK expression and is not toxic to the cells. (*C*) mRNA levels of *APase, bone sialoprotein* (*BSP*), *osteocalcin* (*OC*), *type I collagen* (*Col1A1*), and (*D*) *osterix* and *runx2* in MC3T3-E1 cells transfected with empty vector (Vector), vector containing *ank* cDNA (ANK-WT), or vector containing F376del *ank* cDNA (ANK-Fdel) were determined by real-time PCR and SYBR Green and normalized to 18S RNA levels. Data were obtained from triplicate PCRs using RNA from three different cultures, and values are presented as means ± SD (^a^*p* < .01 versus cells transfected with empty vector; ^b^*p* < .01 versus cells transfected with vector containing wild-type *ank* cDNA). (*E*) MC3T3-E1 cells were cotransfected with a firefly luciferase reporter construct containing six *runx2* DNA-binding elements (pOSE2-luc), a *Renilla* luciferase reporter construct, and either empty vector (Vector), vector containing *ank* cDNA (ANK-WT), or vector containing F376del *ank* cDNA (ANK-Fdel) and cultured for 48 hours after transfection. Cell lysates were analyzed for firefly luciferase activity and normalized to *Renilla* luciferase activity. Data were obtained from three different experiments, and values are presented as means ± SD (^a^*p* < 0.01 versus cells transfected with empty vector; ^b^*p* < .01 versus cells transfected with vector containing wild-type *ank* cDNA).

We first overexpressed wild-type ANK and the F376del CMD mutant ANK in COS cells, which do not normally express ANK. The F376del mutant ANK completely lost its ability to transport intracellular PP_*i*_, as indicated by the same intracellular PP_*i*_ concentration in COS cells transfected with empty vector or vector containing F376del mutant *ank* cDNA (Fig. 5*A*). In contrast, COS cells transfected with vector containing wild-type *ank* cDNA showed a reduced intracellular PP_*i*_ concentration (Fig. 5*A*). Next, we overexpressed wild-type and F376del mutant ANK in the preosteoblastic cell line MC3T3-E1. Cells transfected with expression plasmids encoding wild-type or F376del mutant *ank* showed markedly increased ANK protein expression compared with the levels in cells transfected with empty vector (Fig. 5*B*). Overexpression of wild-type ANK in MC3T3-E1 cells resulted in increased osteoblastogenic differentiation, as indicated by the increased mRNA kevels of bone marker genes, including *APase, BSP, osteocalcin*, and *type I collagen* (Fig. 5*C*). In addition, *osterix* expression was markedly upregulated in ANK-overexpressing MC3T3-E1 cells, whereas *runx2* expression was elevated only slightly (Fig. 5*D*). Overexpression of the F376del mutant form of ANK also resulted in increased mRNA levels of these bone marker genes despite the loss of PP_*i*_ transport activity of the F376del mutant ANK (Fig. 5*C, D*). Contrary to the overexpression of wild-type ANK, overexpression of the F376del mutant ANK resulted in increased mRNA levels of *runx2* (Fig. 5*D*). MC3T3-E1 cells cotransfected with wild-type ANK expression plasmid and the luciferase reporter construct pOSE2 (containing six *runx2* DNA-binding elements) showed increased luciferase activity compared with cells cotransfected with empty vector and the pOSE2 luciferase reporter construct (Fig. 5*E*). Cotransfection of cells with the expression plasmid encoding F376del mutant *ank* and the pOES2 luciferase reporter construct resulted in a further increase in luciferase activity compared with the activity of wild-type ANK-overexpressing cells (Fig. 5*E*).

**Fig. 6:**
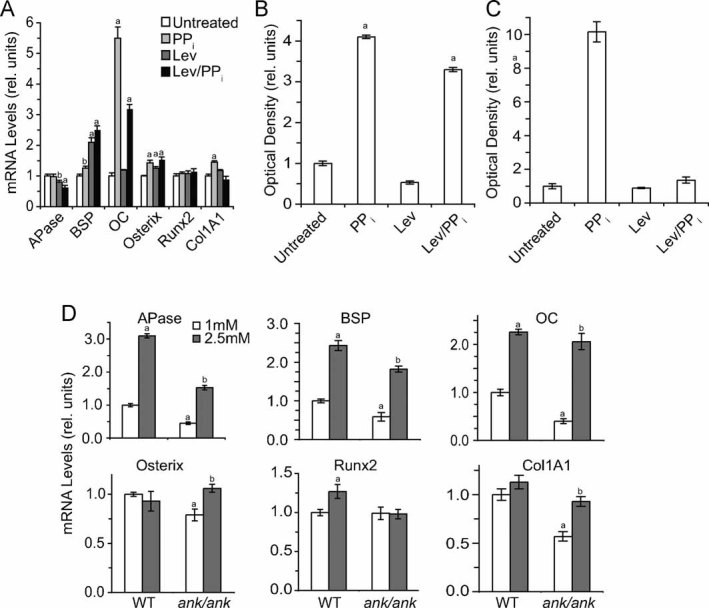
Osteoblastogenic differentiation of MC3T3-E1 or *ank/ank* BMSCs cultured in the presence of PP_*i*_ or P_*i*_. (*A*) MC3T3-E1 cells were cultured for a total of 12 days. The cells were treated for the first 6 days with 0.5 mM PP_*i*_ and/orf levamisole (Lev). mRNA levels of *APase, BSP, osteocalcin* (*OC*), *osterix, runx2*, and *type I collagen* (*Col1A1*) were determined by real-time PCR and SYBR Green and normalized to 18S RNA levels. Data were obtained from triplicate PCRs using RNA from three different cultures, and values are presented as means ± SD (^a^*p* < .01 versus untreated cells; ^b^*p* < .05 versus untreated cells). (*B, C*) MC3T3-E1 cells were cultured for a total of 12 days. The cells were either treated for the first 6 days (*B*) or the last 6 days (*C*) with 0.5 mM PP_*i*_ and/or levamisole. Mineralization was determined by alizarin red S staining. Alizarin red S stain was quantitated as described in Fig. 4 and is expressed as relative units compared with the optical density per milligram of protein of untreated cells, which was set as 1. Data were obtained from four different experiments, and values are presented as means ± SD (^a^*p* < .01 versus untreated cells). (*D*) *ank/ank* and wild-type (WT) BMSCs were cultured in medium containing ascorbate and 1 or 2.5 mM P_*i*_ for 10 days. mRNA levels of *APase, BSP, osteocalcin* (*OC*), *osterix, runx2*, and *type I collagen* (*Col1A1*) were determined by real-time PCR and SYBR Green and normalized to 18S RNA levels. Data were obtained from triplicate PCRs using RNA from three different cultures, and values are presented as means ± SD (^a^*p* < .01 versus wild-type BMSCs cultured in the presence of 1 mM P_*i*_; ^b^*p* < .01 versus *ank/ank* BMSCs cultured in the presence of 1 mM P_*i*_).

Since wild-type ANK regulates extracellular PP_*i*_/P_*i*_ homeostasis, we determined whether extracellular PP_*i*_ regulates osteoblast differentiation and mineralization. We cultured MC3T3-E1 cells in osteoblastogenic differentiation medium in the absence or presence of 0.5 mM PP_*i*_ for the first 6 days of a 12-day culture period. In the presence of PP_*i*_, mRNA levels of *BSP, osteocalcin, osterix*, and *type I collagen* were increased, whereas mRNA levels of *APase* and *runx2* were unchanged or slightly increased (Fig. 6*A*). To determine whether the alterations in bone marker gene expression were directly attributable to PP_*i*_ and not its hydrolysis product P_*i*_, we treated MC3T3-E1 cells with a specific APase inhibitor, levamisole. Levamisole treatment alone resulted in increases in *BSP* and *osterix* mRNA levels and a slight increase of *osteocalcin* mRNA levels, whereas the mRNA levels of the other bone marker genes were slightly decreased compared with untreated cells (Fig. 6*A*). Levamisole/PP_*i*_ treatment showed the same regulatory patterns of bone marker gene mRNA levels as levamisole treatment, with levamisole/PP_i_ treatment being more effective in altering the mRNA levels of these markers (Fig. 6*A*). To determine the effect of extracellular PP_*i*_ and/or P_*i*_ on mineralization, we cultured MC3T3-E1 cells for the first 6 days or the last 6 days of a 12-day culture period with PP_*i*_, levamisole, or levamisole and PP_*i*_ and determined the degree of mineralization by alizarin red S staining after the 12-day culture period. PP_*i*_ and levamisole/PP_*i*_ treatment during the first 6 days of the 12-day culture period enhanced the degree of mineralization of MC3T3-E1 cells, whereas levamisole alone did not change the degree of mineralization (Fig. 6*B*). PP_*i*_ treatment during the last 6 days of the 12-day culture period enhanced mineralization of MC3T3-E1 cells, whereas levamisole or levamisole/PP_*i*_ treatment did not alter the degree of mineralization compared with the mineralization of untreated MC3T3-E1 cells (Fig. 6*C*). These findings suggest that PP_*i*_ directly and P_*i*_ resulting from the hydrolysis of PP_*i*_ stimulate osteoblastogenic differentiation and as a consequence mineralization. In addition, P_*i*_ propagates mineral formation, whereas PP_*i*_ inhibits the propagation of mineralization. Since extracellular P_*i*_ seemed to be more effective in stimulating osteoblastogenic differentiation and mineralization, we determined whether extracellular P_*i*_ can rescue the delayed osteoblastogenic differentiation of *ank/ank* BMSCs. *ank/ank* and wild-type BMSCs were cultured in osteoblastogenic differentiation medium that contains 1 mM P_*i*_ in the absence or presence of additional 1.5 mM extracellular P_*i*_ for 10 days. The additional 1.5 mM P_*i*_ increased the mRNA levels of *APase, BSP, osteocalcin, osterix*, and *type I collagen* of *ank/ank* BMSCs to levels similar to or higher than those of untreated wild-type cells (Fig. 6*D*). And 1.5 mM P_*i*_ treatment of wild-type BMSCs resulted in further increases of *APase, BSP, osteocalcin, runx2*, and *type I collagen* mRNA levels. But 1.5 mM P_*i*_ did not affect the mRNA levels of *osterix* in wild-type BMSCs or *runx2* mRNA levels in *ank/ank* BMSCs (Fig. 6*D*).

### ANK is expressed in osteoclast precursor cells and affects osteoclast differentiation

**Fig. 7:**
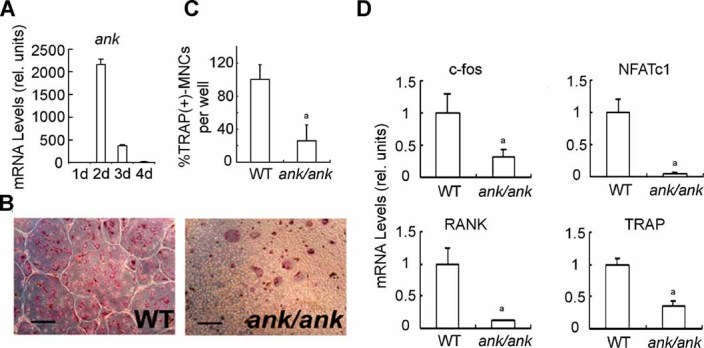
ANK deficiency suppresses osteoclastogenesis in culture. (*A*) *ank* expression during osteoclast differentiation of bone marrow cells. Bone marrow cells were isolated from 5-week-old wild-type mice and cultured in the presence of M-CSF and RANKL for 4 days. Total RNA was isolated after 1, 2, 3, and 4 days of culture. *ank* mRNA levels were determined by real-time PCR analysis using SYBR Green and normalized to 18S RNA levels. Data were obtained from triplicate PCRs using RNA from three different cultures, and values are presented as means ± SD. (*B, C*) Osteoclast development in the culture of bone marrow cells obtained from wild-type (WT) and *ank/ank* mice. TRAP^+^ multinucleated (number of nuclei > 3) cells (MNCs) were induced by M-CSF and RANKL treatment for 6 days. Data were obtained from five different cell cultures, and values are expressed as means ± SD (^a^*p* < .01 versus WT). Bar = 500 µm. (*D*) Real-time PCR analysis of *c-fos, NFATc1, RANK*, and *TRAP*. Real-time PCR analysis was performed using SYBR Green and normalized to the levels of 18S RNA. Data were obtained from triplicate PCRs of three different cultures, and values are presented as means ± SD (^a^*p* < .01 versus WT).

Our observation that ANK function–deficient mice exhibited an osteoclastic phenotype (Fig. 2*E*) prompted us to determine the role of ANK in osteoclastogenesis. When bone marrow cells isolated from wild-type mice were induced to undergo osteoclastic differentiation in vitro by the addition of RANKL and M-CSF, ANK expression was the highest during the initial phase of osteoclast differentiation and decreased in the later stages of osteoclast differentiation (Fig. 7*A*). After 6 days of treatment with RANKL and M-CSF, most wild-type bone marrow cells differentiated into mature osteoclasts (Fig. 7*B*). Osteoclastogenesis in cultures of *ank/ank* bone marrow cells in the presence of M-CSF and RANKL for 6 days was suppressed, as indicated by the reduced number of TRAP^+^ multinucleated cells in these cultures compared with wild-type bone marrow cell cultures (Fig. 7*B, C*). Because osteoclastogenesis is under the control of RANKL/RANK signaling, we analyzed the expression levels of the genes downstream of this axis. The expression level of *c-fos* was decreased in *ank/ank* bone marrow cell cultures treated with M-CSF and RANKL. *NFATc1* expression level, which is a target of *c-fos*, also was decreased in *ank/ank* bone marrow cell cultures compared with wild-type cells (Fig. 7*D*). In addition, *RANK* and *TRAP* mRNA levels were markedly reduced in *ank/ank* bone marrow cell cultures compared with wild-type bone marrow cell cultures (Fig. 7*D*).

## Discussion

Our findings demonstrate that ANK, a transmembrane protein that transports intracellular PP_*i*_ to the extracellular milieu regulates both aspects of bone remodeling: bone formation and bone resorption. Loss of ANK function resulted in delayed osteoblastogenic differentiation of BMSCs, as reflected by reduced numbers of APase^+^ and von Kossa^+^ colonies and reduced expression of bone marker genes, including *APase, BSP, osteocalcin*, and *type I collagen*. In addition, *osterix* expression was decreased in *ank/ank* BMSCs cultured in osteoblastogenic differentiation medium, whereas *runx2* expression was not altered. Conversely, overexpression of ANK resulted in increased osteoblastogenic differentiation of the preosteoblastic cell line MC3T3-E1 and increased *osterix* expression, whereas *runx2* expression was not affected. Overexpression of ANK, however, resulted in stimulation of *runx2* transcriptional activity. *ank/ank* calvarial osteoblasts also showed decreased expression of bone marker genes, including *osterix* and *runx2*. Consequently, the degree of mineralization was decreased during the initiation and early propagation of mineralization of these cells. However, at later stages, mineralization was increased in calvarial osteoblasts lacking ANK function, consistent with the notion that extracellular PP_*i*_ acts as an inhibitor of mineralization via binding to newly formed mineral crystals and preventing their growth.(29) These findings suggest that ANK regulates osteoblast differentiation and mineralization at different levels; ANK is required for osteoblastogenic differentiation into mature osteoblasts, and ANK controls the propagation of osteoblast extracellular matrix mineralization. The extensive hydroxyapatite deposition in articular cartilage and synovial fluid; the spinal, peripheral joint, and ligament bony ankylosis; and the calcification of arteries were attributed to the lack of extracellular PP_*i*_ to inhibit mineral formation in these tissues of *ank/ank* and *ank-*null mice.(12,15) This notion is supported by the findings that nucleotide pyrophosphatase phosphodiesterase 1 (NPP1)–deficient mice show a similar joint phenotype as *ank/ank* mice, and these mice also show arterial calcification.(30,31) NPP1 is an enzyme located on the outer plasma membrane that generates extracellular PP_*i*_ by the hydrolysis of ATP.(32)

Our results show that extracellular PP_*i*_ directly regulates the expression of osteoblast marker genes, including *APase, BSP*, and *osterix*. In addition, treatment of nonmineralized osteoblastic cells with PP_*i*_ even in the presence of the APase-specific inhibitor levamisole resulted in increased mineralization, suggesting that PP_*i*_ directly regulates osteoblastogenic differentiation. In contrast, treatment of osteoblastic cells with PP_*i*_ in the presence of levamisole beginning shortly before the initiation of mineralization inhibited the mineralization process because of the inhibitory role of PP_*i*_ in mineral crystal formation and growth. Therefore, extracellular PP_*i*_ stimulates osteoblastogenic differentiation and, as a result, stimulates the initiation and early propagation of mineralization. Furthermore, extracellular PP_*i*_ controls the propagation and degree of mineralization by regulating mineral crystal growth. Our findings are consistent with a recent finding showing that extracellular PP_*i*_ not only inhibited mineral crystal growth but also directly and independent of its hydrolysis to P_*i*_ regulated osteopontin expression in osteoblasts.(29) Even though no receptor or transport system for extracellular PP_*i*_ is known, the possibility that PP_*i*_ acts through a transmembrane receptor is not unreasonable. Other small molecules also signal through receptors in osteoblasts, including the calcium-sensing receptor,(33) the nucleotide P2-purine receptor,(34) and the bisphosphonates (PP_*i*_ analogues) acting through connexin 43 hemichannels.(35)

In addition, our findings show that extracellular P_*i*_ resulting from the hydrolysis of PP_*i*_ regulates osteoblastogenic differentiation. Extracellular P_*i*_ treatment of *ank/ank* BMSCs resulted in increases in mRNA levels of bone marker genes, including *APase, BSP, osteocalcin, osterix*, and *type I collagen*. In addition, P_*i*_ further increased the mRNA levels of these bone marker genes and *runx2* in wild-type BMSCs. Previous studies demonstrated that P_*i*_ affects cellular differentiation and function of a variety of cells, including chondrocytes, osteoblasts, osteoclasts, and vascular smooth muscle cells.(36–39) The primary mechanism for P_*i*_ entry through the cell membrane is via a family of Na^+^-dependent P_*i*_ transporters.(40,41) Taken together, our findings suggest that ANK-mediated control of osteoblastogenic differentiation and mineralization is mediated by extracellular PP_*i*_/P_*i*_ homeostasis and that the lack of ANK function to regulate this homeostasis results in delayed osteoblastogenic differentiation.

Our findings reveal that ANK also acts as an intrinsic regulator of osteoclast differentiation. The number and surface area of osteoclasts were markedly reduced in *ank/ank* mice. In addition, *ank/ank* precursor cells, when cultured in the presence of RANKL and M-CSF, showed a markedly reduced number of mature osteoclasts after 6-day culture. Our findings also reveal that *ank/ank* osteoblasts show lower *RANKL* and *OPG* mRNA levels than wild-type osteoblasts; however, the *RANKL*/*OPG* ratio was the same. Therefore, it is possible that mature osteoclast numbers are reduced in *ank/ank* mice owing to (1) the loss of ANK function in osteoclast precursors resulting in inhibition of osteoclast differentiation and (2) the reduced levels of RANKL in *ank/ank* osteoblasts resulting in a less effective support of osteoclastogenesis than by wild-type BMSCs and osteoblasts.

Bone formation and bone resorption are two arms of a tightly coupled remodeling process. Imbalance between these two processes can lead to either increased bone mass (osteopetrosis) or decreased bone mass (osteopenia, osteoporosis). Our data indicate that ANK affects both osteoblast and osteoclast differentiation, with the net outcome of reduced bone mass. There are few other examples of a single protein suppressing the differentiation and/or function of both cell types.(42–44) In all cases where a single protein delayed both bone formation and bone resorption, the net outcome was decreased bone mass.(42–44) This outcome was explained by the fact that bone formation is notably slower than bone resorption.

The different phenotypes in cranium (hyperostosis) and long bones (osteopenia) in *ank/ank* mice may result from the different rates of bone remodeling or turnover in craniofacial and long bones. Bone turnover in long bones is much higher than in calvarial bones.(45) Suppression of osteoblast differentiation leads to the formation of immature woven bone, which is easily being resorbed. Therefore, it is possible that the long bone formed in mice lacking ANK function is immature and easily resorbed, resulting in the osteopenic phenotype of the long bones, whereas turnover of craniofacial bone is much lower, eventually resulting in maturation of the immature woven bone and hyperostosis because of the reduced number of functional osteoclasts in *ank/ank* mice. CMD patients and a CMD mouse model show similar but milder phenotypes as *ank/ank* and *ank-*null mice, including hyperostosis of the cranium and osteopenia of the long bones. Our findings revealed that the F376del CMD mutation of ANK stimulated osteoblastogenic differentiation to an even higher degree than wild-type ANK. These findings are consistent with previous studies showing elevated serum APase and TRAP levels in CMD patients and a CMD mouse model, suggesting increased bone formation and bone resorption resulting in increased bone turnover in CMD patients.(13,16,31) The increased bone turnover in long bones explains the osteopenic phenotype in CMD patients and the CMD mouse model, whereas the hyperostosis of the cranium results from the low bone turnover and increased bone formation. Therefore, the similar phenotype of *ank/ank* mice and human CMD patients possibly results from different mechanisms. The F376del CMD mutation integrates into the membrane contrary to the lack of generation of ANK protein in *ank/ank* or *ank*-null mice and stimulates osteoblastogenic differentiation despite its total loss of PP_*i*_ transport activity, suggesting that CMD mutant ANK and wild-type ANK may interact with other intracellular proteins and that these interactions affect signaling pathways and other cellular functions important to the regulation of osteoblast and osteoclast differentiation. The notion that wild-type or mutant forms of ANK may interact with other proteins is supported by a recent study showing the interaction of wild-type ANK with the Na^+^-P_*i*_ cotransporter Pit-1.(46) In addition, previous findings showed that the proline-to-leucine mutation at position 5 (P5L) in ANK cannot stimulate hypertrophic growth plate chondrocyte differentiation as wild-type ANK despite the increased PP_*i*_ transport activity of the P5L mutation compared with wild-type ANK.(47)

In conclusion, our findings provide evidence that ANK plays an important role in osteoblastogenic and osteoclastogenic differentiation and that the lack of ANK function results in delayed osteoblastogenic and osteoclastogenic differentiation. In addition, our findings reveal that ANK regulates osteoblastogenic differentiation by controlling extracellular PP_*i*_/P_*i*_ homeostasis and that PP_*i*_ directly and P_*i*_ as a result of PP_*i*_ hydrolysis are involved in ANK-mediated regulation of osteoblastogenesis. It is possible that ANK-mediated control of extracellular PP_*i*_/P_*i*_ homeostasis also controls osteoclast differentiation because previous studies have shown that extracellular P_*i*_ plays a role in osteoclastogenesis.(39,48) Our findings, however, also suggest that ANK and/or CMD mutant forms of ANK may use interactions with other proteins to regulate osteoblast and osteoclast differentiation. Finally, our findings suggest that ANK regulates bone formation not only during development but also during bone remodeling and therefore may be a novel candidate gene that is important in the development of osteoporosis, a disease characterized by loss of bone mass and strength and often resulting in bone fracture from even minor trauma. Future studies are needed to establish the signaling pathways used by ANK to control these important events during bone remodeling.
